# Endoplasmic reticulum–resident protein Sec62 drives colorectal cancer metastasis via MAPK/ATF2/UCA1 axis

**DOI:** 10.1111/cpr.13253

**Published:** 2022-10-05

**Authors:** Yirong Jin, Yuying Han, Suzhen Yang, Jiayi Cao, Mingzuo Jiang, Jie Liang

**Affiliations:** ^1^ State key Laboratory of Cancer Biology, National Clinical Research Center for Digestive Diseases and Xijing Hospital of Digestive Diseases Air Force Medical University Xi'an China; ^2^ Key Laboratory of Resource Biology and Biotechnology in Western China, Ministry of Education. School of Medicine Northwest University Xi'an China; ^3^ Department of Digestive Disease and Gastrointestinal Motility Research Room The Second Affiliated Hospital of Xian Jiaotong University Xi'an China; ^4^ Department of Gastroenterology and Hepatology Jinling Hospital, Medical School of Nanjing University Nanjing China

## Abstract

**Objective:**

Metastasis is responsible for the poor prognosis of patients with colorectal cancer (CRC), and the role of aberrant expression of endoplasmic reticulum (ER) receptors in tumour metastasis has not been fully elucidated. The aim of the study is to ensure the role of ER‐resident protein Sec62 in CRC metastasis and illuminate associated molecular mechanisms.

**Materials and Methods:**

Bioinformatics analysis, qRT‐PCR, western blot and immunohistochemistry assays were performed to evaluate the expression level and clinical significance of Sec62 in CRC. The specific role of Sec62 in CRC was identified by a series of functional experiments. We conducted RNA sequencing and rescue experiments to analyse the differentially expressed genes and identified UCA1 as a novel pro‐metastasis target of Sec62 in CRC. Besides, the efficacy of MAPK/JNK inhibitor or agonist on Sec62‐mediated CRC metastasis was evaluated by trans‐well and wound healing assays. Finally, luciferase reporter and ChIP assay were employed to further explore the potential mechanisms.

**Results:**

The abnormally elevated expression of Sec62 predicted poor prognosis of CRC patients and facilitated malignant metastasis of CRC cells. Mechanistically, Sec62 enhanced UCA1 expression through activating MAPK/JNK signalling pathway. And the p‐JNK activating ATF2 could transcriptionally regulate UCA1 expression. Furthermore, blocking or activating MAPK/JNK signalling with JNK inhibitor or agonist potently suppressed or enhanced Sec62 mediated CRC metastatic process.

**Conclusions:**

Our study reports for the first time that the Sec62/MAPK/ATF2 /UCA1 axis exists in CRC metastatic process, which could be a potential treatment target of metastatic CRC.

## Graphical abstract

Metastasis accounts for poor prognosis of patients with CRC. The effects of aberrant expression of endoplasmic reticulum (ER) receptors in cancer have not been fully elucidated, especially in CRC. Here, we unveiled that ectopic expression of Sec62 could predict poor prognosis of CRC patients and facilitate malignant metastasis of CRC cells. Mechanistically, Sec62 enhanced UCA1 expression through activating MAPK/ JNK signalling, subsequently potentiated CRC migration and invasion. The p‐JNK activating ATF2 could directly bind to UCA1 promoter and transcriptionally activate UCA1. Clinically, blocking or activating MAPK signalling by inhibitors or agonists potently suppressed or enhanced Sec62‐mediated CRC metastasis. Our study reports the Sec62/MAPK/ATF2 /UCA1 axis may serve as a potential target in metastatic CRC treatment.

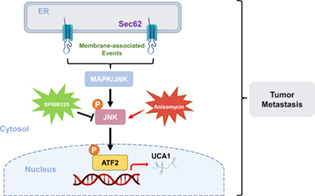

## 1 INTRODUCTION

Colorectal cancer (CRC), despite great advances in diagnostic and therapeutic strategy over the past few decades, remains one of the primary reasons of death caused by malignant tumour worldwide.^1^, ^2^ Metastasis is the key contributor of cancer‐related death, and 5‐year survival rate in patients with stage IV CRC is extremely poor.^3^, ^4^ It is well‐known that tumour metastasis is a multi‐step process that tumour cells develop at the primary site, then progressively disseminate, finally invade distant organs through the gradual accumulation of molecular and phenotypic changes.^5^, ^6^ Remarkable progress has been made in the development of biomarker‐driven targeted therapies for patients with CRC.^7^ Thus, investigations in the aberrant expression of key molecules in CRC metastasis are badly needed to dig out putative targets for metastatic CRC.

SEC62 homologue, preprotein translocation factor (Sec62), a transmembrane protein in endoplasmic reticulum (ER), could transfer nascent synthesized precursor polypeptides into ER in a form of Sec complex and modulate Calcium homeostasis in ER by its C‐terminal EF hand motif.^8^ Recent studies substantiated that the dysfunction of Sec62 frequently occurs in various cancers, which is tightly associated with tumour malignant behaviours. For example, Sec62 has been found to confer resistance on thapsigargin analogs by ameliorating ER stress in prostate cancer.^9^ Sec62 is involved in regulating hepatocellular carcinoma (HCC) aggressiveness by activating integrinα/caveolin1 signalling.^10^ High Sec62 expression indicates a poor clinical prognosis of melanoma patients.^11^ Moreover, Sec62 could activate Wnt/β‐catenin signalling and stimulate chemo‐resistance of CRC cells.^12^ However, functional relevance and potential mechanism of Sec62 in CRC metastasis remain largely unclear.

Here, we report for the first time that Sec62 is upregulated in CRC and accelerates metastasis of CRC cells. Clinically, highly expressed Sec62 predicts a poor prognosis of patient with CRC. Mechanistically, Sec62 reinforces the metastatic potentials of CRC cell via triggering the MAPK/JNK signalling pathway, which enhances the expression of UCA1 by upregulating phospho‐ATF2 (p‐ATF2) level. Overall, our results identify a novel role of Sec62 in CRC metastasis and targeting the Sec62/MAPK/UCA1 axis may provide an effective strategy for CRC treatment.

## 2 METERIALS AND METHODS

### 2.1 Human CRC tissues and cell lines

Fresh CRC and non‐tumour tissues were collected in pairs from patients who received colonic resection in Xijing Hospital, Xi'an, China. Clinical and pathological identifications are correctly labelled as the samples obtained. The study was approved by the Clinical Research Ethics Committee of the Air Force Medical University and Xijing Hospital. Informed consent was obtained from all subjects involved.

Human CRC cell lines (HuTu80, HT29, HCT116, Caco‐2, SW480, SW620, HCT8, LoVo, HCT15, DLD‐1, and SW48) and normal colonic epithelial cells (FHC and NCM460) were cultured in complete DMEM and incubated with 5% CO_2_ at 37 °C. All cell lines were tested and authenticated to ensure that they were not contaminated by bacteria, yeasts, fungi, viruses and mycoplasma and then stored in our laboratory for further research.

### 2.2 Protein extractions and Western blot

RIPA buffer mixed with phosphatase and protease inhibitors (cat# 539134, Merck Millipore, Germany) was used to extract total proteins. SDS‐PAGE gel was used to separate protein aliquots (30–50 μg), following transferred into nitrocellulose filter membranes. Incubation with a primary antibody at 4 °C overnight. In the next day, the protein bands were incubated with secondary antibodies for 1 h. Expression was then assessed using chemiluminescence reagents (Thermo Fisher Scientific). Each blot was scanned by the Molecular Imager ChemiDox XRS+ Imaging System and quantified by the Image Lab software. The relative quantification of the protein was standardized against internal reference (β‐Actin or GAPDH).

Antibodies were employed as follows: anti‐Sec62, anti‐ATF2 (Abcam, USA, ab168843/ab140644, ab32160, respectively), anti‐p38 MAPK, anti‐P‐p38 MAPK, anti‐MAPK/JNK, anti‐P‐MAPK/JNK, anti‐p44/42 MAPK(ERK1/2), anti‐P‐p44/42 MAPK (ERK1/2), anti‐Phospho‐ATF2, anti‐GAPDH and anti‐β‐Actin (Cell Signalling Technology, USA, #8690S, #4511S, #9252S, #4668S, #4695S, #9101S, #40749, #97166S, #8457S, respectively).

### 2.3 RNA extractions and real‐time qPCR

According to the manufacturer's protocol, total RNA was obtained from paired tissues and cultured cells by the RNeasy Kit (cat #74134, Qiagen, Germany). Samples were then reverse‐transcribed into complementary DNA (cDNA) with a PrimeScript RT Reagent Kit (Takara, Japan). Target sequences were amplified using SYBR Green PCR Master Mix (TaKaRa, Japan). β‐Actin and GAPDH were employed as an endogenous control. Relative mRNA level was determined using 2^−ΔΔCt^ method. Detailed sequences information of primers is listed in Table S1.

### 2.4 Immunohistochemical staining

Immunohistochemical (IHC) was conducted using CRC tissue microarray chips, including 100 CRC tissues, 80 paired paraneoplastic tissues, and follow‐up data (Shanghai Biochip, Shanghai, China). The slide was probed with primary anti‐Sec62 antibody (Abcam, USA, ab140644) at 4°C overnight. The sections were incubated with corresponding secondary antibodies. The proteins were visualized with DAB chromogenic substrate, counterstained with haematoxylin. Two pathologists scored the IHC data independently. Staining was evaluated according to histological scoring standards: staining intensity was scored as 0 (no staining), 1 (weak staining), 2 (moderate staining), and 3 (strong staining). The degree of staining was scored based on the percentage of positive cells as follows: 0 (0%), 1 (1–25%), 2 (26%–50%), 3 (51%–75%), and 4 (76%–100%). Staining intensity and degree scores were multiplied to get a final score. A sample with a score of <4 points (0, 1, 2, and 3) was considered as a low expression, and a score of ≥4 points (4, 6, 8, 9, and 12) was identified as having a high expression. Similarly, mice lung tissue sections obtained were stained with P53 antibody (Cell Signalling Technology, USA, #2527S) with IHC analysis. The mean value of these scores was calculated for further statistical analyses.

### 2.5 Plasmid construction

Plasmids were generated as described previously.^13^ The detailed experiment process and sequence information are exhibited in Supporting methods.

### 2.6 Lentivirus production and transduction

The lentivirus was prepared by Shanghai GeneChem. 1 × 10^7^ lentivirus transducing units were used to infect 1 × 10^5^ cells with the presence of 10 ug/ml polybrene. After 48 h, 2.5 μg/ml puromycin was added in the culture medium to screen in the next 2 weeks. Sec62 levels in stably constructed cells were detected with qRT‐PCR and western blot assays, and successfully constructed cells were cultured and saved for subsequent studies.

### 2.7 Transient transfection

For transient transfection, CRC cells (6 × 10^5^ cells per well) were cultured with DMEM with 10% fetal bovine serum for 24 h before experiments, and then cultured with serum‐free medium for another 24 h. Cells were transfected using siRNAs purchased from Ribobio (Guangzhou, China) in DMEM comprising the Attractene Reagent (QIAGEN, Germany), as the manufacturer suggests. The transfected cells were washed with PBS after 6–8 h. At last, the 6‐well plates containing cells were recovered in complete medium for further analysis.

### 2.8 Plasmid infection

The details of experiment process are exhibited in Supporting methods.

### 2.9 In vitro migration and invasion assays

Eight micrometre micropore membrane in 24‐well chambers (Corning Inc., NY, USA) were used to assess invasive and migratory abilities of infected or transfected cells. In invasion assays, Matrigel (Corning, 200 mg/ml) was firstly coated on each chamber insert and dried up overnight. In the next day, the constructed cells (1 × 10^5^ cells) were harvested, resuspended in DMEM medium without serum and then seeded into each upper chamber. The coated chambers and cells were put into 24‐well plates containing DMEM with 20% FBS. Cells were incubated for 24 or 48 h at 37°C. Migration assays have approximately the same procedure of invasion assays, the only difference is non‐coated membrane of chambers used to detect cell migration ability. Each assay was repeated thrice.

### 2.10 Wound healing assay

Wound healing assays were conducted as previous reports.^14^ Briefly, cells were seeded at 6‐well plates until the confluence reached about 90% after 24 h. Then a 200 μl sterile tip was used to scratch gently and slowly across the center of the well to form an artificial gap. The floated cells were discarded and each well was gently washed. The adherent cells were cultured in medium without serum (*t* = 0 h) and imaged at each time point. The gap distances were measured using the Image J software (v1.53c) at these time points to assess cell migration ability. The experiments were repeated independently thrice.

### 2.11 In vivo metastatic model

For in vivo metastasis assays, lentivirus infected CRC cells were evenly suspended in 100 μl of PBS at a density of 1 × 10^6^. The cell suspension is injected to the tail vein of nude mice (female, 6–8 weeks old, *n* = 10/group). And the lungs were surgically obtained to perform histological examination of the lung metastatic nodules 9 weeks later after injections. The animal study was reviewed and approved by the Institutional Animal Care and Use Committee of the Air Force Medical University.

### 2.12 Luciferase reporter assays

The details of Luciferase reporter assays are exhibited in Supporting methods.

### 2.13 Chromatin immunoprecipitation

Pierce Agarose ChIP Kit (Prod #26156, Thermo Scientific™, USA) was used to conduct ChIP assays. Briefly, cells were cross‐linked with 1% formaldehyde and quenched with glycine. The co‐immunoprecipitation was employed to sink the bound DNA from the sonicated cell lysates (8 rounds of 5 s on and 30 s off) with antibodies against ATF2, input controls, and normal IgG (Cell Signalling Technology, Danvers, MA, USA), and subjected to PCR to amplify the corresponding binding sites on the promoters. The primers used in the assay are listed in Table S1.

### 2.14 Agents

The MAPK agonist Anisomycin and inhibitor SP600125 were purchased from Selleck Chemicals (Houston, TX, USA). Cells were treated according to the manufacturer instructions.

### 2.15 Public datasets

Public datasets were obtained from Gene Expression Omnibus (GEO) database, and each dataset was applied for separate usage according to data characteristics, respectively. In details, GSE41258, GSE68468, GSE18105, GSE44076 and GSE41568 were performed to analyse the expression of Sec62 in CRC. CPTAC (https://proteomic.datacommons.cancer.gov/pdc/) was used to obtain Sec62 protein levels in CRC. And GSE44076 and GSE41568 were employed to obtain the mRNA level of UCA1 in CRC.

### 2.16 Quantification and Statistical analysis

SPSS (version 19.0) and Prism (version 8.0) were utilized to analyse all the statistical data. Data is presented as mean ± standard deviation (SD). Chi‐square test of independence was employed to analyse categorical data. Two‐group comparisons of the quantitative data were analysed with the Student's *t*‐test. Statistical analyses gathering over two groups were performed using ANOVA followed by intragroup comparisons with *p* value adjustments. The correlation of two independent variables was analysed by Spearman correlation analysis. Normality test for the variable data was performed before Student's *t*‐test and ANOVA. Kaplan–Meier survival analysis and the log‐rank test were used to determine the cumulative survival rates. *p* < 0.05 represents statistical significance.

## 3 RESULTS

### 3.1 Sec62 is upregulated in colorectal cancer and predicts poor overall survival

**FIGURE 1:**
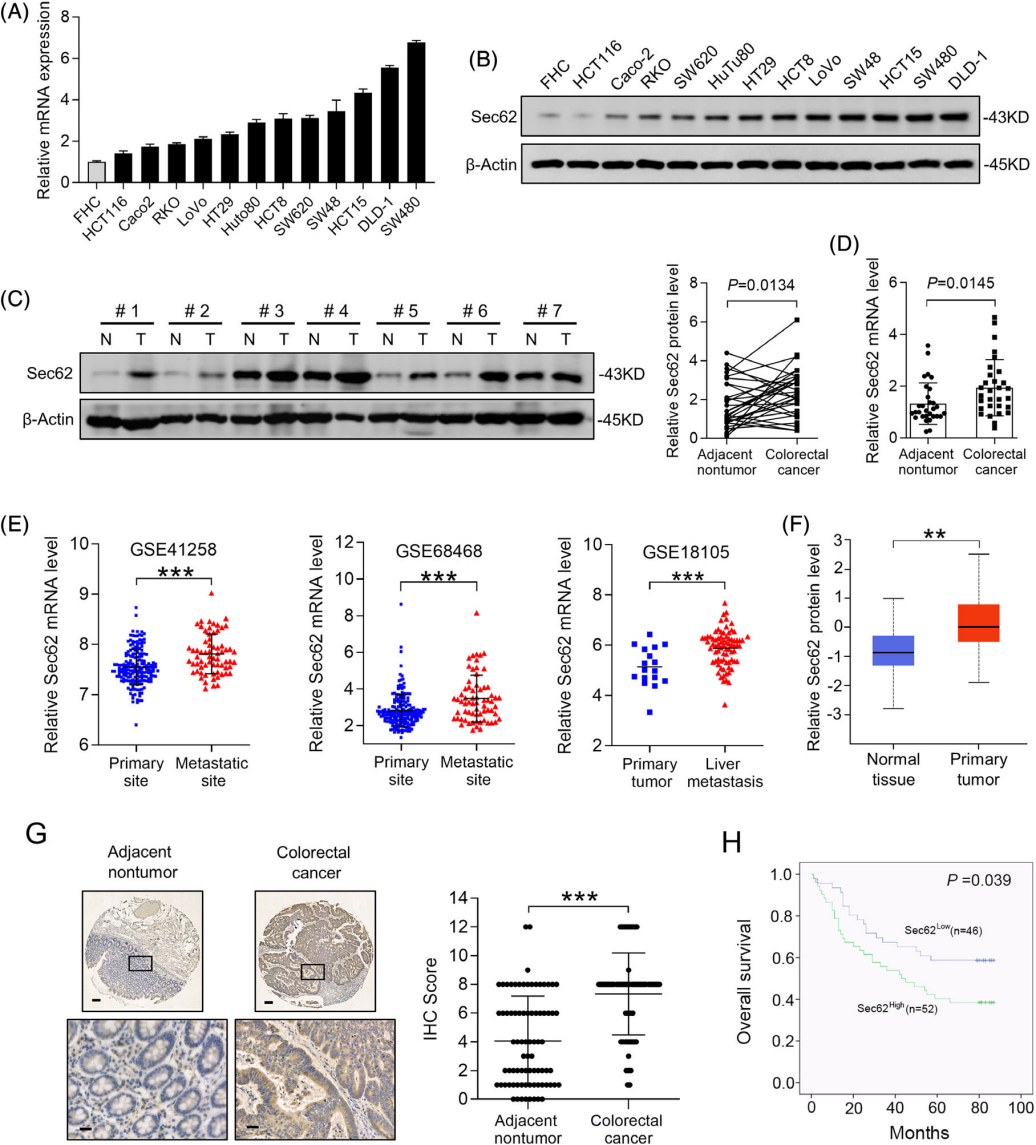
Sec62 is upregulated in colorectal cancer (CRC) and predicts poor overall survival. (A and B) qRT‐PCR and western blot, respectively shownthe mRNA and protein level of Sec62 in different CRC cell lines (*n* = 12) and FHC, an immortalized colonic epithelial cell line. (C and D) The expression of Sec62 in paired primary CRC tissues (*T*, *n* = 30) and adjacent non‐tumour samples (*N*, *n* = 30) was determined by Western blot and qRT‐PCR analysis. (E) The transcriptional level of Sec62in the CRC primary site and metastatic site was identified from GEO database (GSE41258, GSE68468, GSE18105). (F) CPTAC database was used to detect the protein level of Sec62 between CRC tissues and normal colon tissues. (G) Representative positive immunohistochemical (IHC) staining for Sec62 of paired CRC (*n* = 100) and adjacent non‐tumour samples (*n* = 80) (left). The semiquantitative analysis evaluated the scores of Sec62 expression in samples (right). Scale bars, 100 μm (up) or 20 μm (below). (H) Kaplan–Meier analysis exhibited the different overall survival (OS) time in patients with CRC with different Sec62 level. ***p < 0.001, **p < 0.01 and *p < 0.05. Each assay was repeated thrice (*N* ≥ 3), Data are presented as mean ± standard deviation (SD)

To gain insight into the function of Sec62 in CRC progression, we first evaluated Sec62 expression profile in cell lines with qRT‐PCR and western blot assays and found it was upregulated in CRC cells (Figure 1A,B). Next, paired CRC and non‐tumour clinical samples were used to evaluate the level of Sec62 by qRT‐PCR and western blot. Consistently, Sec62 was highly expressed in CRC compared with non‐tumour tissues (Figure 1C,D). Moreover, Gene Expression Omnibus (GEO) database (GSE68468,^15^, ^16^, ^17^, ^18^, ^19^, ^20^, ^21^ GSE41258^21^, ^22^ and GSE18105^23^) also exhibited CRC metastatic sites contained higher Sec62 mRNA level than primary sites (Figure 1E). And the high protein level of Sec62 was also shown in CRC tissues after analysing UALCAN database^24^ (Figure 1F).

Next, we focused on the clinical significance of Sec62 in CRC. Immunohistochemistry (IHC) was conducted and Sec62 expression was quantified in a cohort of CRC patients. Consistent with a previous study,^12^ IHC analysis verified that compared to nontumor tissues, Sec62 protein was higher in CRC samples. (Figure 1G, Figure S1A). Correlation analysis revealed that Sec62 positively related with the larger tumour size, the more lymph node metastasis, and the higher AJCC stage (Table 1). In addition, Kaplan–Meier survival analysis demonstrated that CRC patients with elevated expression of Sec62 indicated a much worse prognosis than those with low Sec62 expression (Figure 1H).

**TABLE 1:** The correlation between SEC62 levels and clinicopathologic features of patients with colorectal cancer

Characteristics	Number of case	Expression of sec62		*p* value
		Low	High	
Gender				0.069
Male	58	32	26	
Female	41	15	27	
Age				0.303
<60	39	21	18	
≥60	60	25	35	
Pathology grade				0.514
I‐II	70	32	38	
III‐IV	30	14	16	
Tumour size				**0.03**
≤5 cm	36	22	14	
>5 cm	64	24	40	
Depth of invasion				0.198
T2–T3	68	28	40	
T4	32	18	14	
Lymph node metastasis				**0.017**
No	52	30	22	
Yes	48	16	32	
AJCC stage				**0.010**
1–2	51	30	21	
3–4	49	16	33	

*Note*: The bold values represent *p* < 0.05.

Taken together, all these results suggested that Sec62 was upregulated in CRC, and its expression positively correlated with the prognosis of CRC patients.

### 3.2 Sec62 facilitates CRC metastasis in vitro and in vivo

**FIGURE 2:**
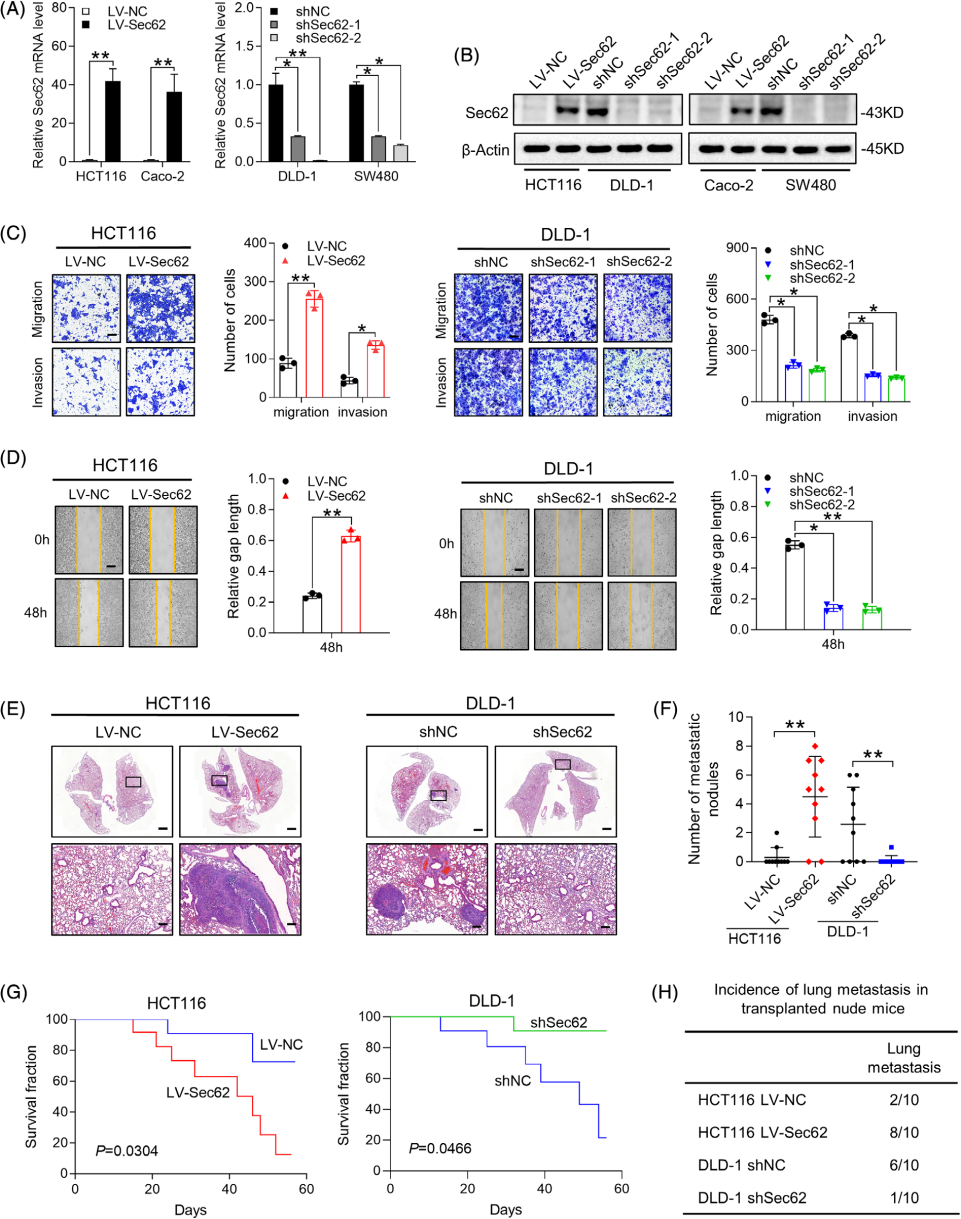
Sec62 facilitates CRC metastasis in vitro and in vivo. (A and B) CRC cells were separately transfected with overexpressed or knockdown Sec62 lentivirus, and harvested for qRT‐PCR and western blot to validate the lentivirus transfection efficiency. (C and D) Metastatic abilities of CRC cells after lentivirus transfection were measured by trans‐well (C) and wound healing (D) assays. The percentage of wound gap was normalized to 0 h. Scale bars, 10 μm. (E) Representative H&E staining of mice lung tissue sections resected from each group after injection of instructed CRC cells 8 weeks later (*n* = 10 per group). Scale bars: 250 μm (up) and 25 μm (below). (F) The numbers of surface lung metastatic foci were calculated in different groups. (G) Overall survival (OS) of the nude mice of each group. (H) Statistical analysis for incidences of vivo metastases from indicated groups. *p < 0.05 and **p < 0.01. *N* ≥ 3, Data are presented as mean ± SD

According to the results of Figure 1A,B, we firstly overexpressed Sec62 by transduction of lentiviral Sec62 (LV‐Sec62) in HCT116 and Caco‐2 cells, which had low endogenous Sec62 level. Knockdown of Sec62 used two shRNAs (shSec62‐1, ‐2) in DLD‐1 and SW480 cells. Lentivirus transfection efficiency was assured by qRT‐PCR and western blot (Figure 2A,B).

Next, trans‐well assay was performed to assess metastatic potentials of constructed CRC cells, and the results indicated that Sec62 upregulation markedly increased CRC motility, while Sec62 downregulation significantly reduced the malignant invasion of CRC (Figure 2C and Figure S1C). Likewise, wound healing assay also determined the promotion effects of Sec62 on CRC cell migration and invasion (Figure 2D and Figure S1D). Besides, we built an in vivo metastatic animal model to study the effects of Sec62 in CRC. As expected, overexpressed Sec62 increased the number of metastatic lung nodules (Figure 2E,F and Figure S1B), enhanced the incidence of lung metastasis, (Figure 2H) and resulted in the shortened survival time of mice (Figure 2G, left). On the contrary, Sec62 suppression led to an opposite result (Figure 2E,F, Figure 2G, right and Figure S1B). All these data pointed out that Sec62 functioned as a pro‐metastatic oncogene in CRC migration and invasion.

### 3.3 UCA1 is indispensable for Sec62‐mediated CRC cell metastasis

**FIGURE 3:**
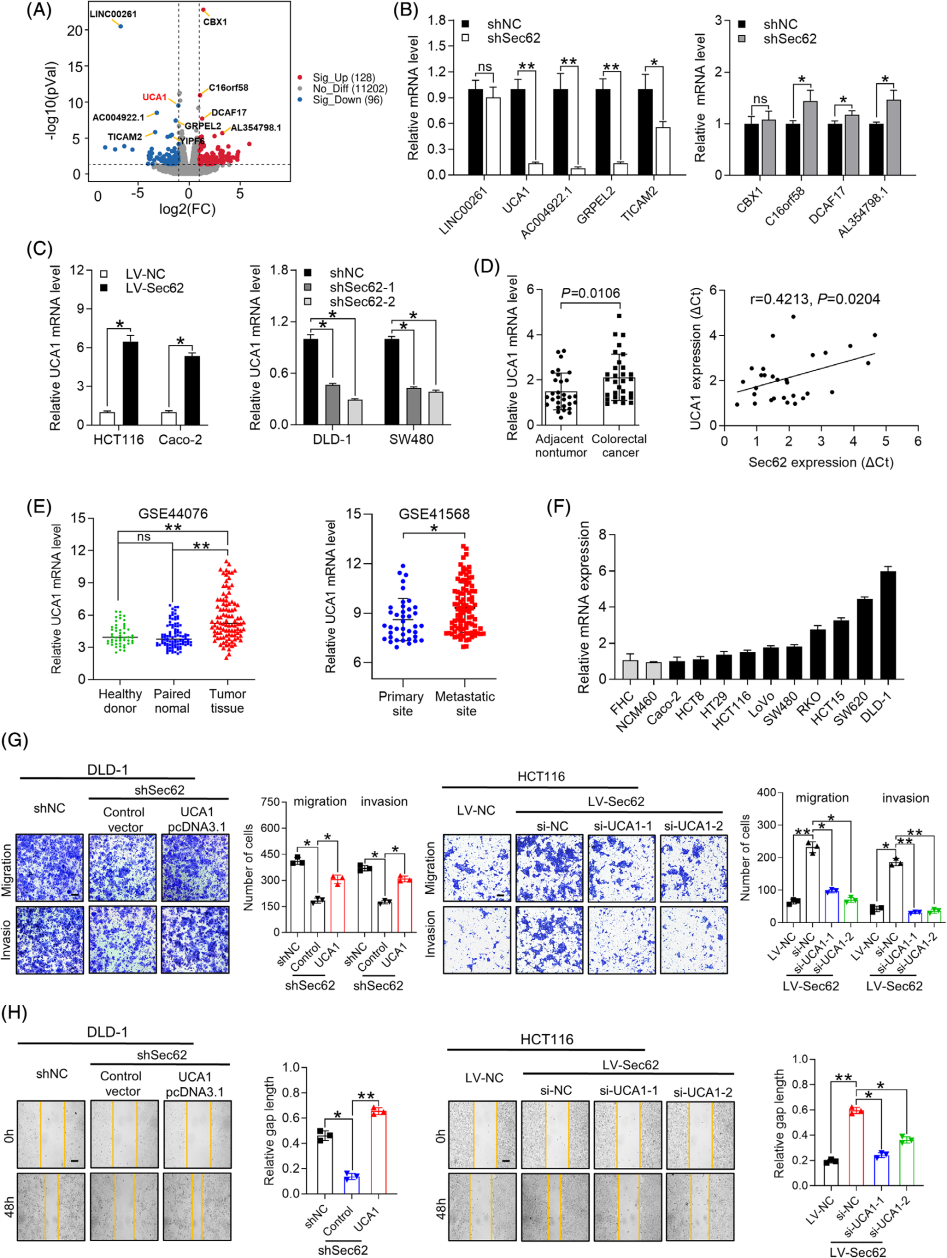
UCA1 is indispensable for Sec62‐mediated CRC cell metastasis. (A) Volcano map exhibited differentially expressed genes when Sec62 was inhibited in DLD‐1 cells (Sig‐UP: significant up‐regulation; No‐Diff: no difference; Sig‐Down: significant down‐regulation). (B) qRT‐PCR analysis of obvious changed candidate genes in Sec62‐silencing DLD‐1 cells. (C) The expression of UCA1 was detected by qRT‐PCR in Sec62 upregulated or downregulated CRC cells. (D) The level of UCA1 in paired CRC tissues and non‐tumour tissues was determined by qRT‐PCR (Left, *n* = 30). A positive correlation between the mRNA level of Sec62 and UCA1 in CRC tissues was identified (right, *n* = 30). (E) GEO datasets was utilized to analyse UCA1 transcriptional level among healthy donor colon tissues (healthy donor, *n* = 50), paired non‐tumour normal colon tissues (paired normal, *n* = 98) and CRC tissues (tumour tissue, *n* = 98) (left, GSE44076); and the level of UCA1 in primary sites (*n* = 39) and metastatic sites (*n* = 94) was measured from GSE41568 (right). (F) UCA1 expression in CRC cell lines (*n* = 10) and two immortalized colon epithelial cell lines, FHC and NCM460 was identified by qRT‐PCR. (G and H) Trans‐well (G) and wound healing (H) assays shown the motility ability of Sec62 silencing DLD‐1 cells after transfection with plasmid of UCA1 (left, Control = control vector, UCA1 = UCA1 pcDNA3.1) and Sec62 overexpressing HCT116 cells transfected with of siRNA UCA1 (right). The percentage of wound gap was normalized to 0 h. Scale bars, 10 μm. **p < 0.01 and *p < 0.05, ns = No Significance. *N* ≥ 3. Data are presented as mean ± SD

In order to further dig out the Sec62‐related mechanisms in CRC metastasis, RNA sequencing (RNA seq) was carried out to determine the downstream effector. One hundred twenty‐eight genes were significantly upregulated and 96 genes were downregulated (fold change >2 and *p* < 0.05) when Sec62 was suppressed (Figure 3A). As shown in Figure 3B and S2A, we screened several obviously changed candidate genes to validate the reliability of RNA seq analysis.

Based on the results of previous researches,^25^, ^26^, ^27^ TCGA analysis and RNA seq, UCA1 has been tightly linked to CRC malignancy. We examined the expression of UCA1 in Sec62 knockdown or overexpression cells (Figure 3C) and CRC clinical samples (Figure 3D, left). Expression of UCA1 consistently changed with Sec62 knockdown or overexpression and among CRC tissues there existed a positive correlation between Sec62 and UCA1 expression (Figure 3D, right). Similar to the expression patterns of Sec62, UCA1 was verified to be increased in CRC tissues (GSE44076,^28^, ^29^, ^30^, ^31^, ^32^, ^33^, ^34^ Figure 3D,E, left), which are further augmented in CRC metastatic site (GSE41568,^35^ Figure 3E, right). Compared with NCM460 and FHC, UCA1 was significantly elevated in CRC cells (Figure 3F). Moreover, the promotive effects of UCA1 in CRC metastasis was proven by trans‐well and wound healing assays (Figure S2B,C). All the results supported that UCA1 is a promising functional target of Sec62 and worth to be further studied.

Subsequent rescue experiments confirmed that Sec62 promote CRC metastasis in an UCA1‐dependent manner. Specifically, both trans‐well and wound healing assays demonstrated that UCA1 downregulation partially impaired Sec62‐mediated cell motility, while upregulated UCA1 restored the metastatic ability of Sec62‐knockdown CRC cells (Figure 3G,H and Figure S2D,E). Collectively, we deduced that UCA1 was not only crucial in CRC metastasis, but also served as an indispensable functional target of Sec62.

### 3.4 Sec62 induces UCA1 expression via MAPK signalling pathway

**FIGURE 4:**
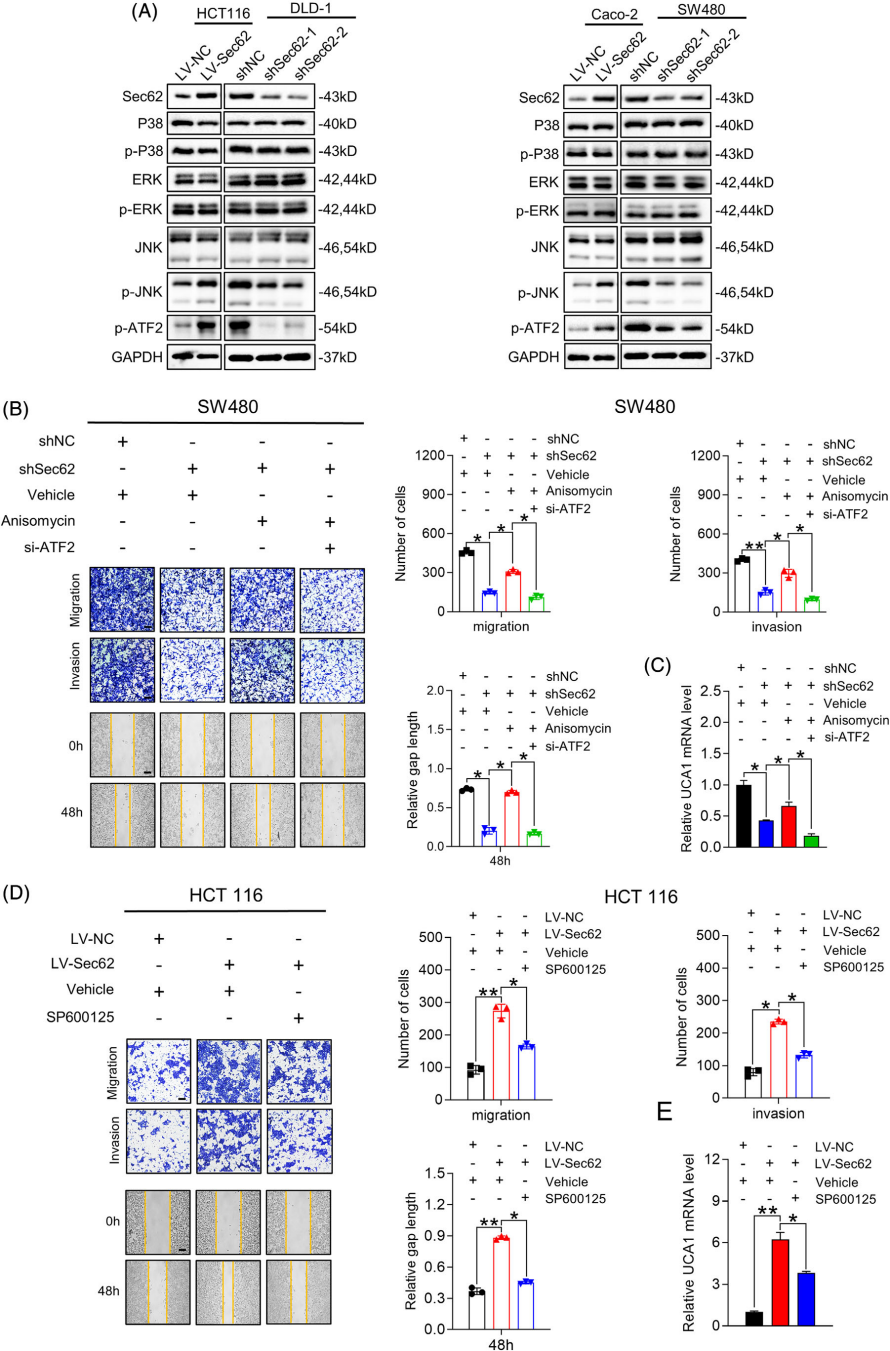
Sec62 induces UCA1 expression via MAPK signalling pathway. (A) In Sec62 overexpressing and knockdown cell models, total and phosphorylation of P38, ERK, JNK and p‐ATF2 protein expression were detected by western blot. (B) SW480 cell was transfected with Sec62 shRNA or shNC lentivirus and then treated with JNK agonist Anisomycin or ATF2 siRNA, the metastatic capability of cells was determined by trans‐well (up) and wound‐healing assays (below). The percentage of wound gap was normalized to 0 h. Scale bars, 10 μm. (C) qRT‐PCR outcomes of UCA1 levels in the indicated cells. (D) Trans‐well (up) and wound healing (below) assays measured the metastatic ability of HCT116 cells transfected with LV‐Sec62 or LV‐NC lentivirus and then treated with JNK inhibitor SP600125. The percentage of wound gap was normalized to 0 h. Scale bars, 10 μm. (E) qRT‐PCR analysis of UCA1 expression in the indicated cells. **p < 0.01 and *p < 0.05, *N* ≥ 3. Data are presented as mean ± SD

Sec62 has been found to regulate various cellular biological processes by triggering diverse signalling pathways.^36^ And it has been reported that UCA1 expression could be upregulated by the activation of MAPK signalling.^26^, ^37^ The activity of the MAPK pathway is self‐limiting under physiological conditions, accomplished by rapid self‐phosphorylation and upstream inhibition.^38^, ^39^, ^40^ However, it kept continuous activated status in abnormal cases, and consequently propelling tumour progression.^41^ Therefore, we speculated that Sec62 might mediate UCA1 expression via regulating the MAPK signalling. MAPK signalling is often studied by its major subfamilies, containing JNK, ERK, and p38, and their activated forms (p‐JNK, p‐ERK, and p‐p38).^39^ To validate this hypothesis, we employed western blot to detect the protein changes of MAPK downstream kinases and found that overexpressed Sec62 remarkably increased the level of phosphor‐JNK (p‐JNK), while p38 and ERK phosphorylation were still being unchanged (Figure 4A). These results clued that Sec62 could activate the MAPK/JNK signalling pathway.

To solidify the result that MAPK/JNK signalling could be triggered by Sec62 activation in CRC, we measured whether activating or suppressing MAPK/JNK signalling with JNK agonist (Anisomycin) or inhibitor (SP600125) would affect Sec62‐mediated CRC metastatic capability. In vitro metastatic assays showed that activation of the MAPK/JNK signalling by JNK agonist (Anisomycin) partly recovered CRC cell mobility and upregulated UCA1 expression in Sec62‐silencing cells. (Figure 4B,C). On the contrary, after treated with JNK inhibitor (SP600125), the aggressive ability of CRC and UCA1 expression in LV‐Sec62 cells were remarkably suppressed (Figure 4D,E). All these results demonstrated that MAPK/JNK signalling was functionally taking part in Sec62‐mediated CRC metastasis, and targeting MAPK/JNK pathway by JNK inhibitors might be helpful for Sec62 overexpressing CRC metastasis.

### 3.5 ATF2 as a transcription factor activated by the MAPK/JNK pathway regulates the expression of UCA1

**FIGURE 5:**
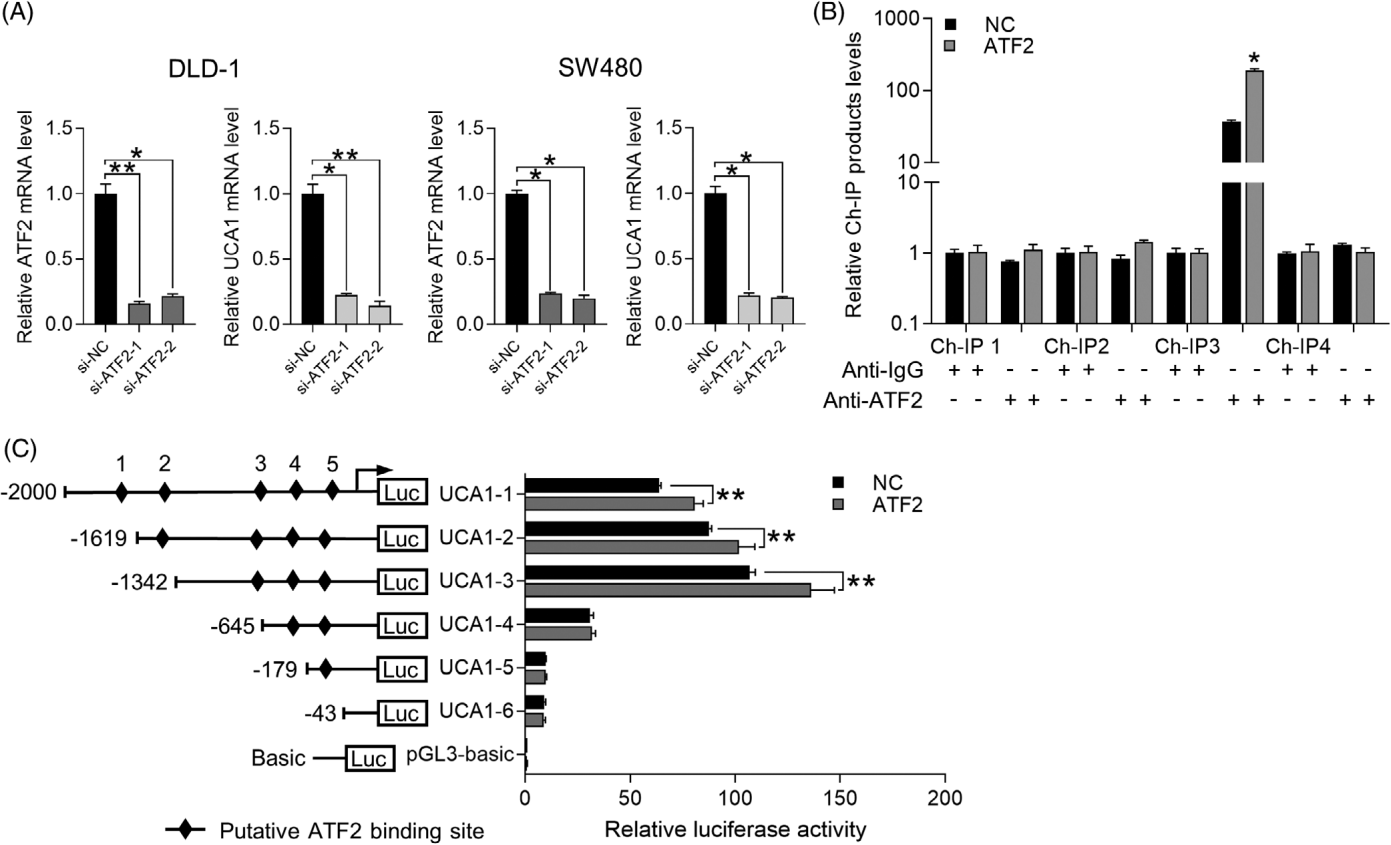
ATF2 as a transcription factor activated by the MAPK/JNK pathway regulates the expression of UCA1. (A) Transcriptional level change of ATF2 and UCA1 in colorectal cells treated with ATF2 siRNA. (B) ChIP assays demonstrated the direct binding of ATF2 at the UCA1 upstream promoter region about ‐895 bp to ‐640 bp. Data are presented as the relative enrichment normalized to control IgG. (C) A schematic representation of consecutive deletion constructed spanning the −2000 to +263 region of the UCA1 promoter region. The putative ATF2‐binding sites in the UCA1 promoter are shown as the black diamond. The luciferase vector pGL3 driven by full length or deletion UCA1 promoter was transfected in DLD‐1 cells, and luciferase activity was measured. Luciferase values are normalized to the empty vector control. **p < 0.01, *p < 0.05, ns = No Significance. *N* ≥ 3, Data are presented as mean ± SD.

ATF2, a crucial MAPK downstream transcription factor, has been proven to be activated by JNK signal pathway.^42^ In our study, p‐JNK activating ATF2 exhibited a significant change in response to Sec62 upregulation or downregulation, implying that p‐ATF2 might be regulated by Sec62 (Figure 4A). Previous studies have reported that UCA1 and ATF2 showed a positive correlation in prostate cancer.^43^ Similarly, in this study, we found that downregulation of ATF2 obviously reduced UCA1 expression in DLD‐1 and SW480 cells (Figure 5A). And the accelerated effects of MAPK/JNK signalling on CRC metastasis could be interrupted by ATF2 suppression (Figure 4B,C).

Considering the molecular characteristics of ATF2 and UCA1, we decided to study whether transcription factor ATF2 could bind to the promoter of lncRNA UCA1. We successfully constructed UCA1 promotor pGL3‐Basic‐full fragment reporter plasmid (UCA1‐1) and five UCA1 pGL3‐Basic‐truncated fragment reporter plasmid (UCA1‐2‐6). Functional analysis of dual‐luciferase assay system in DLD‐1 cells exhibited that the changed promoter activity of UCA1‐1, 2, 3 was significant. As shown in Figure 5C, five potential ATF2 binding sites existed within 2 kilobase(kb) upstream of the UCA1 transcription start site and UCA1‐3 (−1342 bp to −645 bp) was measured as the ATF2‐regulated dominant binding site for UCA1 transcriptional activation (Figure 5C). Furthermore, ChIP assays directly proved that ATF2 could bind to Ch‐IP 3 fragment (−895 bp to −640 bp) in DLD‐1 cells (Figure 5B). The above results all concluded that Sec62 increased UCA1 expression through JNK‐mediated ATF2 activation.

**FIGURE 6:**
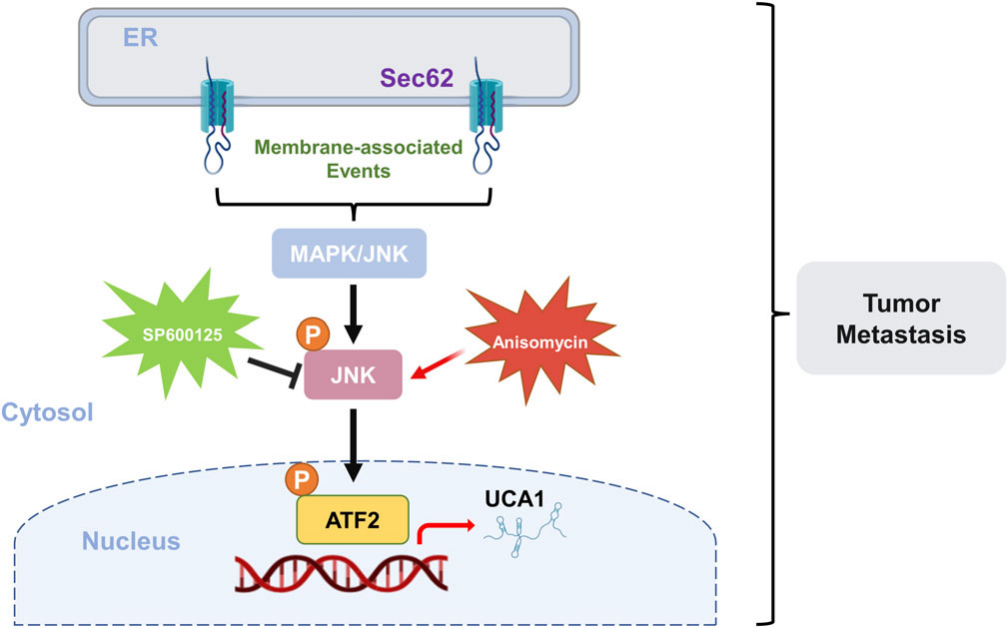
Schematic illustration of the Sec62/MAPK/ATF2 /UCA1 axis in metastatic colorectal cancer cells

In conclusion, we identified ER‐resident protein Sec62 could promote CRC metastasis through a MAPK/ATF2/UCA1 functional axis in this study (Figure 6).

## 4 DISCUSSION

Only a few studies researched the role of ER receptors in malignant cancer development, not mention to explore its relevant underlying mechanisms so far. In this study, we firstly discovered that ER‐resident Sec62 was generally upregulated in CRC using CRC cell lines, clinical samples and public databases, which was positively associated with tumour size, AJCC stage, lymph node metastasis rate and patients' poor prognosis. Next, Sec62 up‐ or down‐regulated cells were constructed to carried out gain‐ or loss‐ functional experiments, demonstrating that Sec62 serve as an oncogene in CRC metastasis. By conducting RNA‐seq, qRT‐PCR and rescue experiments, UCA1 was selected as a downstream target of Sec62. And MAPK/JNK pathway was testified to be triggered by Sec62 and then participated in the regulation of UCA1 by ATF2. Moreover, intervention of MAPK/JNK signalling by JNK inhibitor or agonist made efficient impacts on Sec62 mediated cell metastatic capability. Our study is the first to report that Sec62 may be able to accelerate CRC motility and possess potential therapeutic values in metastatic CRC.

Although several studies towards the role of Sec62 have mentioned it serves as an oncogene in malignant cancer, such as prostate cancer,^44^, ^45^ non‐small‐cell lung cancer,^46^ thyroid cancer,^47^ cervical cancer cells,^48^ and human embryonic kidney cells,^49^ only a few have explored relevant underlying mechanisms. In this study, we discovered a complete novel Sec62/MAPK/ATF2/UCA1 regulatory axis existed in CRC, which enriched the role of Sec62 in CRC and cleared the potential mechanisms associated. Moreover, it is well‐known that UCA1 is widely upregulated in tumours, which could contribute to cancer biological progression, including proliferation,^50^ metastasis,^51^ drug resistance^52^ and tumour angiogenesis.^53^ But the reasons for its overexpression in tumours are rarely studied.^54^ Herein, we identified that the upregulation of UCA1 resulted from Sec62 overexpression. Besides, evidence accumulated that Sec62 substantially contributes to cancer cell malignancy by activating diverse signalling pathways.^36^ And there are growing number of researchers begin to explore the distinct role of MAPK in cancer development, such as metastasis,^55^ drug resistance^56^ and tumour immunotherapy.^57^, ^58^ Here, our data elucidated Sec62 may trigger MAPK/JNK signalling as a bridge to connect Sec62 and UCA1 in CRC metastasis. ATF2, a canonical transcription factor belonged to MAPK/JNK signalling, was confirmed to activate UCA1 by directly binding to its promoter region here. In another study, however, UCA1 was confirmed may function as a ceRNA to reversely regulate ATF2 expression in prostate cancer,^43^ which is an interesting phenomenon worth further investigating.

However, there are some questions remaining unsolved in our study. Although Sec62 was determined to be overexpressed in CRC, why its upregulation occurred had not been clearly answered. Finding potential reasons inducing Sec62 dysfunction in CRC may create significant clinical values for CRC patients. Second, we merely observed phospho‐JNK altered with the change of Sec62 and verified JNK agonist and inhibitor had effects on CRC metastasis, but specific mechanisms of how Sec62 activated the MAPK/JNK pathway was still not elucidated. Thirdly, our results only indicated that Sec62 was associated with several pathologic parameters of CRC, however, Sec62 could act as a potential clinical biomarker that needs validation among larger cohorts in the further.

Growing numbers of researcher begin to turn their attention to Sec62 recently.^12^, ^59^, ^60^ Focusing on the promoting metastatic role of Sec62, we obtained some meaningful hints that Sec62 may be involved in metastasis progression in three major ways. First, as the cytosolic Ca^2+^ level crucially influences cell migration processes^61^ that are seriously disturbed by an uncontrolled Ca^2+^ efflux from the ER.^62^ The passive Ca^2+^ efflux through the Sec61 channel is partly regulated by Sec62.^63^ This clued that Sec62 may regulate tumour metastasis via influencing calcium homeostasis in ER. Second, it is a highly conserved process for precursor protein and peptide to be transported across ER membrane in eukaryotic cell and Sec62 could modulate this process in the form of Sec complex.^64^ Therefore, we could reasonably assume that abnormality in metastasis‐associated precursor protein depending on Sec62 trafficking may occur during tumour metastasis. Furthermore, Sec62 plays a critical role in eukaryotic recovery from ER stress condition, which was described as recovER‐phagy process. Similarly, it is possible that Sec62 mediated recovER‐phagy and then regulated metastasis.^36^ All those suggested that there may be more complex contexts of Sec62 in CRC metastasis and the regulatory axis that Sec62/JNK/ATF2/UCA1 discovered here is a tip of the iceberg. Further investigations still deserved to be complemented in the future.

## AUTHOR CONTRIBUTION

Yirong Jin, Yuying Han and Suzhen Yang: conceptualization and design; acquisition of data; analysis and interpretation of data; manuscript drafting. Yirong Jin, Yuying Han and Jiayi Cao: experiment performance. Suzhen Yang, Jiayi Cao and Yuying Han: data analysis and materials. Yirong Jin: manuscript writing. Mingzuo Jiang and Jie Liang: supervision of all study. All authors read and approved the final manuscript.

## CONFLICT OF INTEREST

No competing interests exist.

## Supporting information


**APPENDIX S1** Supporting Information

**TABLE S1** Primer sequences used in the study

**FIGURE S1** Sec62 facilitates CRC metastasis in vitro and in vivo. (A) Representative H&E staining of paired adjacent non‐tumour (*n* = 100) and primary CRC tissues (*n* = 80). Scale bars: 100 μm (up) and 20 μm (below). (B) Representative IHC staining of p53 in mice resected lung tissue samples from different groups (*n* = 10 per group). Scale bars: 100 μm (up) and 20 μm (below). (C and D) Trans‐well (up) and wound‐healing (below) assays exhibited the metastatic capacity in Caco‐2 transfected with LV‐Sec62 or LV‐NC lentivirus and SW480 cells infected by Sec62 shRNA or shNC lentivirus, respectively. The percentage of wound closure was normalized to 0 h. Scale bars, 10 μm. **p < 0.01, *p < 0.05. *N* ≥ 3, Data are presented as mean ± SD

**FIGURE S2** UCA1 is indispensable for Sec62‐mediated CRC cell metastasis. (A) qRT‐PCR analysis of candidate genes in Sec62 overexpressing HCT116 cells. (B and C) Trans‐well (B) and wound healing (C) assays detected the migratory and invasive abilities of cells transfected with UCA1 siRNA. (D and E) Trans‐well (D) and Wound healing (E) assays determined that the metastatic potentials of Sec62 silencing SW480 cells transfected with UCA1 plasmid and Sec62 overexpressing Caco‐2 cells transfected with UCA1 siRNA. Scale bars, 10 μm. The percentage of wound closure was normalized to 0 h. Scale bars, 10 μm. **p < 0.01, *p < 0.05, ns = No Significance. *N* ≥ 3, Data are presented as mean ± SD

## Data Availability

The data that support the findings of this study are available from the corresponding author upon reasonable request. RNA seq data has been uploaded to Sequence Read Archive (SRA): https://trace.ncbi.nlm.nih.gov/Traces/sra/?study=SRP350689.

## References

[1] Sung H , Ferlay J , Siegel RL , et al. Global cancer statistics 2020: GLOBOCAN estimates of incidence and mortality worldwide for 36 cancers in 185 countries. CA Cancer J Clin. 2021;71(3):209‐249. 33538338 10.3322/caac.21660

[2] von Karstedt S , Walczak H . An unexpected turn of fortune: targeting TRAIL‐Rs in KRAS‐driven cancer. Cell Death Discovery. 2020;6:14. 32194994 10.1038/s41420-020-0249-4 PMC7078304

[3] Siegel RL , Miller KD , Goding Sauer A , et al. Colorectal cancer statistics, 2020. CA Cancer J Clin. 2020;70(3):145‐164. 32133645 10.3322/caac.21601

[4] van der Stok EP , Spaander MCW , Grünhagen DJ , Verhoef C , Kuipers EJ . Surveillance after curative treatment for colorectal cancer. Nat Rev Clin Oncol. 2017;14(5):297‐315. 27995949 10.1038/nrclinonc.2016.199

[5] Birkbak NJ , McGranahan N . Cancer genome evolutionary trajectories in metastasis. Cancer Cell. 2020;37(1):8‐19. 31935374 10.1016/j.ccell.2019.12.004

[6] Lambert AW , Pattabiraman DR , Weinberg RA . Emerging biological principles of metastasis. Cell. 2017;168(4):670‐691. 28187288 10.1016/j.cell.2016.11.037 PMC5308465

[7] Di Nicolantonio F , Vitiello PP , Marsoni S , et al. Precision oncology in metastatic colorectal cancer ‐ from biology to medicine. Nat Rev Clin Oncol. 2021;18(8):506‐525. 33864051 10.1038/s41571-021-00495-z

[8] Crowley KS , Liao S , Worrell VE , Reinhart GD , Johnson AE . Secretory proteins move through the endoplasmic reticulum membrane via an aqueous, gated pore. Cell. 1994;78(3):461‐471. 8062388 10.1016/0092-8674(94)90424-3

[9] Greiner M , Kreutzer B , Lang S , et al. Sec62 protein level is crucial for the ER stress tolerance of prostate cancer. Prostate. 2011;71(10):1074‐1083. 21557272 10.1002/pros.21324

[10] Du J , Zhao Z , Zhao H , et al. Sec62 promotes early recurrence of hepatocellular carcinoma through activating integrinα/CAV1 signalling. Oncogenesis. 2019;8(12):74. 31822656 10.1038/s41389-019-0183-6 PMC6904485

[11] Muller CSL , Pfohler C , Wahl M , et al. Expression of SEC62 oncogene in benign, malignant and borderline melanocytic tumors‐unmasking the wolf in Sheep's clothing? Cancers (Basel). 2021;13(7):1645. 33915997 10.3390/cancers13071645 PMC8036965

[12] Liu X , Su K , Sun X , et al. Sec62 promotes stemness and chemoresistance of human colorectal cancer through activating Wnt/β‐catenin pathway. J Exp Clin Cancer Res. 2021;40(1):132. 33858476 10.1186/s13046-021-01934-6 PMC8051072

[13] Liu H , Du F , Sun L , et al. GATA6 suppresses migration and metastasis by regulating the miR‐520b/CREB1 axis in gastric cancer. Cell Death Dis. 2019;10(2):35. 30674866 10.1038/s41419-018-1270-x PMC6426848

[14] Liu J , Han P , Gong J , et al. Knockdown of KIAA1199 attenuates growth and metastasis of hepatocellular carcinoma. Cell Death Discovery. 2018;4:102. 30455988 10.1038/s41420-018-0099-5 PMC6232158

[15] Getz G , Gal H , Kela I , Notterman DA , Domany E . Coupled two‐way clustering analysis of breast cancer and colon cancer gene expression data. Bioinformatics. 2003;19(9):1079‐1089. 12801868 10.1093/bioinformatics/btf876

[16] Gavert N , Sheffer M , Raveh S , et al. Expression of L1‐CAM and ADAM10 in human colon cancer cells induces metastasis. Cancer Res. 2007;67(16):7703‐7712. 17699774 10.1158/0008-5472.CAN-07-0991

[17] Hertzberg L , Betts DR , Raimondi SC , et al. Prediction of chromosomal aneuploidy from gene expression data. Genes Chromosomes Cancer. 2007;46(1):75‐86. 17044051 10.1002/gcc.20391

[18] Tsafrir D , Bacolod M , Selvanayagam Z , et al. Relationship of gene expression and chromosomal abnormalities in colorectal cancer. Cancer Res. 2006;66(4):2129‐2137. 16489013 10.1158/0008-5472.CAN-05-2569

[19] Puca R , Nardinocchi L , Gal H , et al. Reversible dysfunction of wild‐type p53 following homeodomain‐interacting protein kinase‐2 knockdown. Cancer Res. 2008;68(10):3707‐3714. 18483253 10.1158/0008-5472.CAN-07-6776

[20] Tsafrir D , Tsafrir I , Ein‐Dor L , Zuk O , Notterman DA , Domany E . Sorting points into neighborhoods (SPIN): data analysis and visualization by ordering distance matrices. Bioinformatics. 2005;21(10):2301‐2308. 15722375 10.1093/bioinformatics/bti329

[21] Sheffer M , Bacolod MD , Zuk O , et al. Association of survival and disease progression with chromosomal instability: a genomic exploration of colorectal cancer. Proc Natl Acad Sci U S A. 2009;106(17):7131‐7136. 19359472 10.1073/pnas.0902232106 PMC2678450

[22] Martin ML , Zeng Z , Adileh M , et al. Logarithmic expansion of LGR5(+) cells in human colorectal cancer. Cell Signal. 2018;42:97‐105. 28958617 10.1016/j.cellsig.2017.09.018 PMC5766032

[23] Matsuyama T , Ishikawa T , Mogushi K , et al. MUC12 mRNA expression is an independent marker of prognosis in stage II and stage III colorectal cancer. Int J Cancer. 2010;127(10):2292‐2299. 20162577 10.1002/ijc.25256

[24] Chandrashekar DS , Bashel B , Balasubramanya SAH , et al. UALCAN: a portal for facilitating tumor subgroup gene expression and survival analyses. Neoplasia. 2017;19(8):649‐658. 28732212 10.1016/j.neo.2017.05.002 PMC5516091

[25] Luan Y , Li X , Luan Y , et al. Circulating lncRNA UCA1 promotes malignancy of colorectal cancer via the miR‐143/MYO6 Axis. Molecular Therapy Nucleic Acids. 2020;19:790‐803. 31955010 10.1016/j.omtn.2019.12.009 PMC6970172

[26] Barbagallo C , Brex D , Caponnetto A , et al. LncRNA UCA1, upregulated in CRC biopsies and downregulated in serum exosomes, controls mRNA expression by RNA‐RNA interactions. Molecular Therapy Nucleic Acids. 2018;12:229‐241. 30195762 10.1016/j.omtn.2018.05.009 PMC6023947

[27] Neve B , Jonckheere N , Vincent A , Van Seuningen I . Epigenetic regulation by lncRNAs: an overview focused on UCA1 in colorectal cancer. Cancers (Basel). 2018;10(11):440. 30441811 10.3390/cancers10110440 PMC6266399

[28] Sole X , Crous‐Bou M , Cordero D , et al. Discovery and validation of new potential biomarkers for early detection of colon cancer. PLoS One. 2014;9(9):e106748. 25215506 10.1371/journal.pone.0106748 PMC4162553

[29] Cordero D , Sole X , Crous‐Bou M , et al. Large differences in global transcriptional regulatory programs of normal and tumor colon cells. BMC Cancer. 2014;14:708. 25253512 10.1186/1471-2407-14-708 PMC4182786

[30] Sanz‐Pamplona R , Berenguer A , Cordero D , et al. Aberrant gene expression in mucosa adjacent to tumor reveals a molecular crosstalk in colon cancer. Mol Cancer. 2014;13:46. 24597571 10.1186/1476-4598-13-46 PMC4023701

[31] Closa A , Cordero D , Sanz‐Pamplona R , et al. Identification of candidate susceptibility genes for colorectal cancer through eQTL analysis. Carcinogenesis. 2014;35(9):2039‐2046. 24760461 10.1093/carcin/bgu092 PMC4146415

[32] Berdiel‐Acer M , Sanz‐Pamplona R , Calon A , et al. Differences between CAFs and their paired NCF from adjacent colonic mucosa reveal functional heterogeneity of CAFs, providing prognostic information. Mol Oncol. 2014;8(7):1290‐1305. 24839936 10.1016/j.molonc.2014.04.006 PMC5528579

[33] Moreno V , Alonso MH , Closa A , et al. Colon‐specific eQTL analysis to inform on functional SNPs. Br J Cancer. 2018;119(8):971‐977. 30283144 10.1038/s41416-018-0018-9 PMC6203735

[34] Diez‐Villanueva A , Jorda M , Carreras‐Torres R , et al. Identifying causal models between genetically regulated methylation patterns and gene expression in healthy colon tissue. Clin Epigenetics. 2021;13(1):162. 34419169 10.1186/s13148-021-01148-9 PMC8380335

[35] Lu M , Zessin AS , Glover W , Hsu DS . Activation of the mTOR pathway by Oxaliplatin in the treatment of colorectal cancer liver metastasis. PLoS One. 2017;12(1):e0169439. 28060954 10.1371/journal.pone.0169439 PMC5218497

[36] Linxweiler M , Schick B , Zimmermann R . Let's talk about secs: Sec61, Sec62 and Sec63 in signal transduction, oncology and personalized medicine. Signal Transduct Target Ther. 2017;2:17002. 29263911 10.1038/sigtrans.2017.2 PMC5661625

[37] Liu Z , Wang Y , Yuan S , et al. Regulatory role of long non‐coding RNA UCA1 in signaling pathways and its clinical applications. Oncol Lett. 2021;21(5):404. 33777227 10.3892/ol.2021.12665 PMC7988699

[38] Rovida E , Stecca B . Mitogen‐activated protein kinases and hedgehog‐GLI signaling in cancer: a crosstalk providing therapeutic opportunities? Semin Cancer Biol. 2015;35:154‐167. 26292171 10.1016/j.semcancer.2015.08.003

[39] Drosten M , Barbacid M . Targeting the MAPK pathway in KRAS‐driven tumors. Cancer Cell. 2020;37(4):543‐550. 32289276 10.1016/j.ccell.2020.03.013

[40] Kyriakis JM , Avruch J . Mammalian MAPK signal transduction pathways activated by stress and inflammation: a 10‐year update. Physiol Rev. 2012;92(2):689‐737. 22535895 10.1152/physrev.00028.2011

[41] Fang JY , Richardson BC . The MAPK signalling pathways and colorectal cancer. Lancet Oncol. 2005;6(5):322‐327. 15863380 10.1016/S1470-2045(05)70168-6

[42] Gupta S , Campbell D , Dérijard B , Davis RJ . Transcription factor ATF2 regulation by the JNK signal transduction pathway. Science (New York, NY). 1995;267(5196):389‐393. 10.1126/science.7824938 7824938

[43] Zhang S , Dong X , Ji T , Chen G , Shan L . Long non‐coding RNA UCA1 promotes cell progression by acting as a competing endogenous RNA of ATF2 in prostate cancer. Am J Transl Res. 2017;9(2):366‐375. 28337266 PMC5340673

[44] Yu Y , Gao F , He Q , Li G , Ding G . lncRNA UCA1 functions as a ceRNA to promote prostate cancer progression via sponging miR143. Mol Ther Nucleic Acids. 2020;19:751‐758. 31954329 10.1016/j.omtn.2019.11.021 PMC6962633

[45] Zhao X , Wang Y , He J , et al. LncRNA UCA1 maintains the low‐tumorigenic and nonmetastatic status by stabilizing E‐cadherin in primary prostate cancer cells. Mol Carcinog. 2020;59(10):1174‐1187. 32805084 10.1002/mc.23247

[46] Nie W , Ge HJ , Yang XQ , et al. LncRNA‐UCA1 exerts oncogenic functions in non‐small cell lung cancer by targeting miR‐193a‐3p. Cancer Lett. 2016;371(1):99‐106. 26655272 10.1016/j.canlet.2015.11.024

[47] Wang X , Zhang Y , Zheng J , Yao C , Lu X . LncRNA UCA1 attenuated the killing effect of cytotoxic CD8 + T cells on anaplastic thyroid carcinoma via miR‐148a/PD‐L1 pathway. Cancer Immunol Immunother. 2021;70(8):2235‐2245. 33486611 10.1007/s00262-020-02753-y PMC10992874

[48] Yan Q , Tian Y , Hao F . Downregulation of lncRNA UCA1 inhibits proliferation and invasion of cervical cancer cells through miR‐206 expression. Oncol Res. 2018. doi:10.3727/096504018X15185714083446 29523226

[49] Li Y , Wang T , Li Y , et al. Identification of long‐non coding RNA UCA1 as an oncogene in renal cell carcinoma. Mol Med Rep. 2016;13(4):3326‐3334. 26935146 10.3892/mmr.2016.4894

[50] Liu J , Luo C , Zhang C , et al. Upregulated lncRNA UCA1 inhibits trophoblast cell invasion and proliferation by downregulating JAK2. J Cell Physiol. 2020;235(10):7410‐7419. 32067230 10.1002/jcp.29643

[51] Gong P , Qiao F , Wu H , et al. LncRNA UCA1 promotes tumor metastasis by inducing miR‐203/ZEB2 axis in gastric cancer. Cell Death Dis. 2018;9(12):1158. 30464170 10.1038/s41419-018-1170-0 PMC6249325

[52] Pan J , Li X , Wu W , et al. Long non‐coding RNA UCA1 promotes cisplatin/gemcitabine resistance through CREB modulating miR‐196a‐5p in bladder cancer cells. Cancer Lett. 2016;382(1):64‐76. 27591936 10.1016/j.canlet.2016.08.015

[53] Guo Z , Wang X , Yang Y , et al. Hypoxic tumor‐derived Exosomal long noncoding RNA UCA1 promotes angiogenesis via miR‐96‐5p/AMOTL2 in pancreatic cancer. Mol Ther Nucleic Acids. 2020;22:179‐195. 32942233 10.1016/j.omtn.2020.08.021 PMC7498711

[54] Hosseini NF , Manoochehri H , Khoei SG , Sheykhhasan M . The functional role of long non‐coding RNA UCA1 in human multiple cancers: a review study. Curr Mol Med. 2021;21(2):96‐110. 32560605 10.2174/1566524020666200619124543

[55] Urosevic J , Garcia‐Albéniz X , Planet E , et al. Colon cancer cells colonize the lung from established liver metastases through p38 MAPK signalling and PTHLH. Nat Cell Biol. 2014;16(7):685‐694. 24880666 10.1038/ncb2977

[56] Ahronian LG , Sennott EM , Van Allen EM , et al. Clinical acquired resistance to RAF inhibitor combinations in BRAF‐mutant colorectal cancer through MAPK pathway alterations. Cancer Discov. 2015;5(4):358‐367. 25673644 10.1158/2159-8290.CD-14-1518 PMC4390490

[57] Smith MP , Sanchez‐Laorden B , O'Brien K , et al. The immune microenvironment confers resistance to MAPK pathway inhibitors through macrophage‐derived TNFα. Cancer Discov. 2014;4(10):1214‐1229. 25256614 10.1158/2159-8290.CD-13-1007 PMC4184867

[58] Ebert PJR , Cheung J , Yang Y , et al. MAP kinase inhibition promotes T cell and anti‐tumor activity in combination with PD‐L1 checkpoint blockade. Immunity. 2016;44(3):609‐621. 26944201 10.1016/j.immuni.2016.01.024

[59] Su S , Shi YT , Chu Y , et al. Sec62 promotes gastric cancer metastasis through mediating UPR‐induced autophagy activation. Cell Mol Life Sci. 2022;79(2):133. 35165763 10.1007/s00018-022-04143-2 PMC11073224

[60] Meng Y , Zhao H , Zhao Z , Yin Z , Chen Z , Du J . Sec62 promotes pro‐angiogenesis of hepatocellular carcinoma cells under hypoxia. Cell Biochem Biophys. 2021;79(4):747‐755. 34120320 10.1007/s12013-021-01008-6

[61] Huang JB , Kindzelskii AL , Clark AJ , Petty HR . Identification of channels promoting calcium spikes and waves in HT1080 tumor cells: their apparent roles in cell motility and invasion. Cancer Res. 2004;64(7):2482‐2489. 15059902 10.1158/0008-5472.can-03-3501

[62] Berridge MJ . The endoplasmic reticulum: a multifunctional signaling organelle. Cell Calcium. 2002;32(5–6):235‐249. 12543086 10.1016/s0143416002001823

[63] Linxweiler M , Schorr S , Schäuble N , et al. Targeting cell migration and the endoplasmic reticulum stress response with calmodulin antagonists: a clinically tested small molecule phenocopy of SEC62 gene silencing in human tumor cells. BMC Cancer. 2013;13:574. 24304694 10.1186/1471-2407-13-574 PMC3878975

[64] Jung SJ , Kim H . Emerging view on the molecular functions of Sec62 and Sec63 in protein translocation. Int J Mol Sci. 2021;22(23):12757. 34884562 10.3390/ijms222312757 PMC8657602

