# Supplementary material for: Endoplasmic reticulum–resident protein Sec62 drives colorectal cancer metastasis via MAPK/ATF2/UCA1 axis
Source: Cell Prolif. 2022 Oct 5;55(12):e13253. doi: 10.1111/cpr.13253 (PMC9715360; doi:10.1111/cpr.13253)
Supplement: Supplementary file 1 — APPENDIX S1 Supporting Information [file CPR-55-e13253-s004.docx]

**Endoplasmic Reticulum-Resident Protein Sec62 drives Colorectal Cancer Metastasis by upregulating UCA1 via MAPK Signaling Pathway**

**SUPPLEMENTARY MATERIALS AND METHODS**

**1 |  Plasmid construction**

The UCA1 promoter construct was generated as described previously [15]. Briefly, (−2000/ + 263) UCA1 was generated from human genomic DNA. This construct, corresponding to the sequence from −2000 to +263 (relative to the transcriptional start site) of the 5′- flanking region of the human gene, was generated with forward and reverse primers incorporating NheIF and HindIII sites at the 5′ and 3′ ends, respectively. The NheIF and HindIII sites of the PGL-3 Basic Vector (Promega) were inserted into the final PCR product. Constructs including a deletion of the 5′-flanking regions of the UCA1 promoter (−1619/+263) UCA1, (−1342/+263) UCA1, (−645/ + 263) UCA1, (-179/ + 263) UCA1) and (-43/ + 263) UCA1) were generated in a manner analogous to that for the (−2000/+263) UCA1 construct. All constructs were subsequently validated by sequencing.

**2 |  Plasmid infection**

Cells were prepared in 6-well plates until the convergence reached 75%. The old medium was discarded and cells were washed twice with PBS. 1 ml of serum-free medium was added into each well firstly. 4 µg plasmid (UCA1 pcDNA3.1 or pcDNA3.1-vector) (Genepharma) and 5 µl Lipofectamine™ 2000 (Invitrogen) were mixed and incubated together for 15 min. Then the above mixture was added into wells. After culturing with cells for 4–6 h, medium was then replaced by 2 ml complete medium. And cells were screened with 2.5 μg/ml puromycin (OriGene, MD, USA).

**3 |  Luciferase reporter assays**

Briefly, the mentioned cells were co-transfected with the pGL3-UCA1 promoter fragment, pRL-SV40 Renilla luciferase reporter, and ATF2 plasmid or negative control. Lysis buffer was added to lyse the transfected cells, and the resulting lysate was centrifuged for 1 min in an Eppendorf microcentrifuge at maximum speed. Renilla and rirefly luciferase activities were detected using a dual-luciferase assay (Promega). Renilla luciferase activity was normalized to the firefly activity and presented as relative luciferase activity. Relative luciferase activity was measured using a Modulus TM TD20/20 luminometer (Turner Biosystems, USA).
