# Supplementary material for: Endoplasmic reticulum–resident protein Sec62 drives colorectal cancer metastasis via MAPK/ATF2/UCA1 axis
Source: Cell Prolif. 2022 Oct 5;55(12):e13253. doi: 10.1111/cpr.13253 (PMC9715360; doi:10.1111/cpr.13253)
Supplement: Supplementary file 2 — TABLE S1 Primer sequences used in the study [file CPR-55-e13253-s001.docx]

**Supplementary table 1. Primer sequences used in the study.**

| Primer name | Primer sequence | Enzyme | |
| --- | --- | --- | --- |
| Primers for real-time PCR: |  | |  |
| Sec62 sense: | 5’- TCAGTGTGGGTGCAGGCTGTTT -3’ | |  |
| Sec62 antisense: | 5’- ACCAAAAGTGGTGCCTTCCTCC -3’ | |  |
| ATF2 sense: | 5’- CAATCCACTGCCATGGCCTT -3’ | |  |
| ATF2 antisense: | 5’- TCAGATAAAGCCAAGTCGAATCTGG -3’ | |  |
| UCA1 sense | 5’- CACTCTTTGCCAGCCTCAGCTT -3’ | |  |
| UCA1 antisense | 5’- AGGTGTGAGTGGCGGTCTGAAT -3’ | |  |
| β-actin sense: | 5’- GGTCATCACCATTGGCAA -3’ | |  |
| β-actin antisense: | 5’- GAGTTGAAGGTAGTTTCGTGGA -3’ | |  |
| Primers for UCA1 promoter construct: | | |  |
| (-2000/+263) UCA1 sense: | 5’- ctagctagcTCTATTTTATAGAATGGGATGCTTCTTCGG -3’ | | NheIF |
| (-1619/+263) UCA1 sense: | 5’- ctagctagcTCTCAGGTCACTCCAACCTCTGCCCGCCCT -3’ | | NheIF |
| (-1342/+263) UCA1 sense: | 5’- ctagctagcGTGTGAGGGTTTCGGCTCTCGAGTCAAGAT -3’ | | NheIF |
| (-645/+263) UCA1 sense: | 5’- ctagctagcAGTTCGAGACCAGCCTGGCAAACATGGTGA -3’ | | NheIF |
| (-179/263) UCA1 sense: | 5’- ctagctagcCTAGGCAATGTAGTGAGACCCTGACTCTAT -3’ | | NheIF |
| (-43/263) UCA1 sense: | 5’- ctagctagcCTATATAACCTCAGACATGCCCCAAACCCT -3’ | | NheIF |
| Antisense: | 5’- cccaagcttCCGCCTCCGGCCTGGGGATCCTGTGGGAGG -3’ | | HindIII |
| Primers used for ChIP with the UCA1 promoter: | |  | |
| binding site 1 sense: | 5’- ATTGATGGGTATTGGATTTATG -3’ |  | |
| binding site 1 antisense: | 5’- GGGTGCCTGTAATCCCAGCTAC -3’ |  | |
| binding sites 2 sense: | 5’- GTAGCTGGGATTACAGGTTTC -3’ |  | |
| binding sites 2 antisense: | 5’- AACCCTCACACAGTGACTTC -3’ |  | |
| binding site 3 sense: | 5’- TCGCTGCAGCCATAATGGAAG -3’ |  | |
| binding site 3 antisense: | 5’- GAACTTCTGACCTCAAATGATC -3’ |  | |
| binding site 4 sense: | 5’- AGTTTGAGGTCAGCCTAGGC -3’ |  | |
| binding site 4 antisense: | 5’- AGGATGACCTGATCTGACAC -3’ |  | |
| Sequence of lentivirus: | |  | |
| shRNA-Sec62-1 | 5’- CTGTGGTTGACTACTGCAAC -3’ |  | |
| shRNA-Sec62-2 | 5’- ACAGTTGAATCGAAGATACT -3’ |  | |
| ShRNA-control | 5’- CGCTTCCGCGGCCCGTTCAA -3’ |  | |
