# Supplementary figures and images for: Endoplasmic reticulum–resident protein Sec62 drives colorectal cancer metastasis via MAPK/ATF2/UCA1 axis
Source: Cell Prolif. 2022 Oct 5;55(12):e13253. doi: 10.1111/cpr.13253 (PMC9715360; doi:10.1111/cpr.13253)

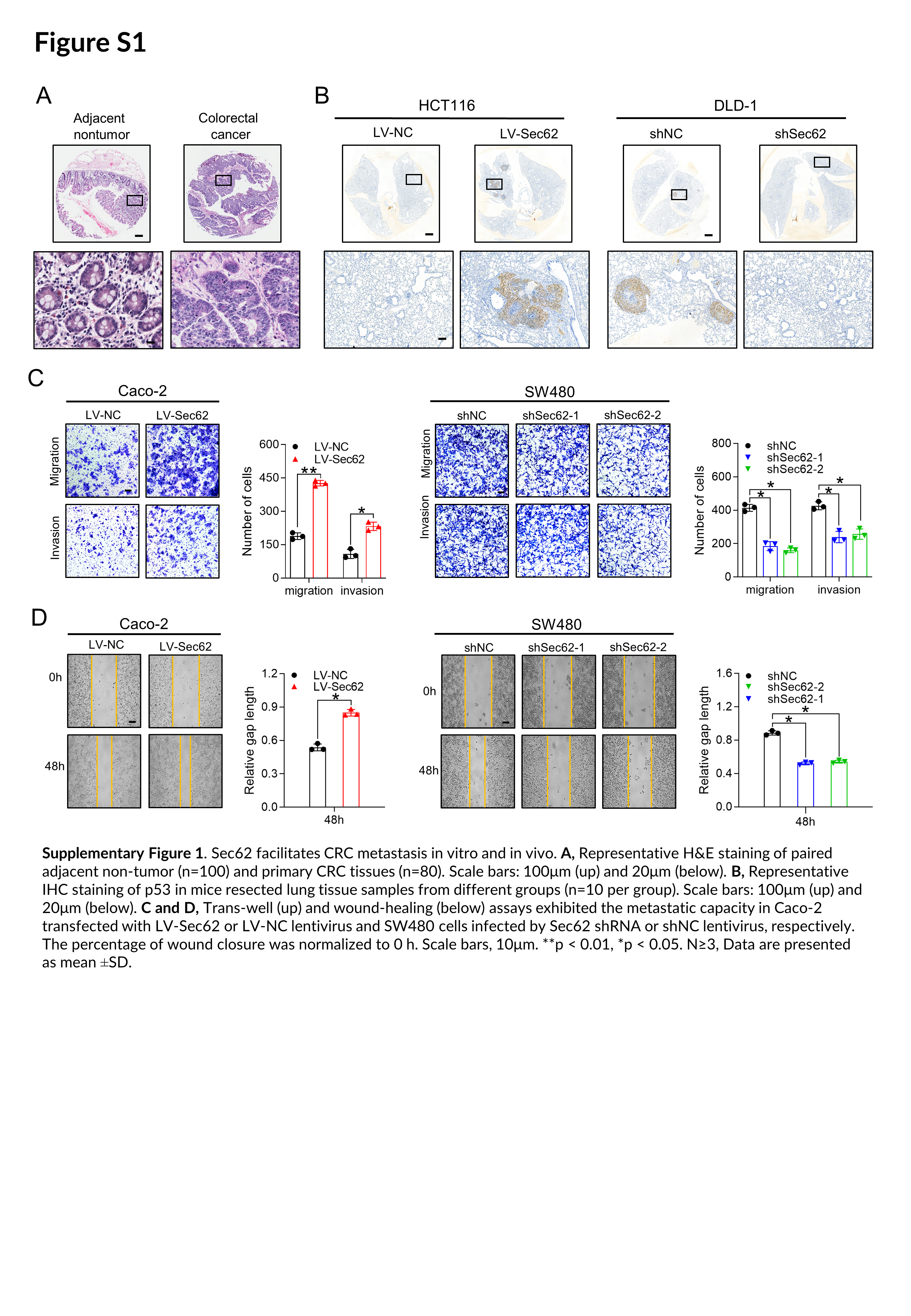

Supplement: Supplementary file 3 — FIGURE S1 Sec62 facilitates CRC metastasis in vitro and in vivo. (A) Representative H&E staining of paired adjacent non‐tumour (n = 100) and primary CRC tissues (n = 80). Scale bars: 100 μm (up) and 20 μm (below). (B) Representative IHC staining of p53 in mice resected lung tissue samples from different groups (n = 10 per group). Scale bars: 100 μm (up) and 20 μm (below). (C and D) Trans‐well (up) and wound‐healing (below) assays exhibited the metastatic capacity in Caco‐2 transfected with LV‐Sec62 or LV‐NC lentivirus and SW480 cells infected by Sec62 shRNA or shNC lentivirus, respectively. The percentage of wound closure was normalized to 0 h. Scale bars, 10 μm. **p < 0.01, *p < 0.05. N ≥ 3, Data are presented as mean ± SD [file CPR-55-e13253-s003.tif]

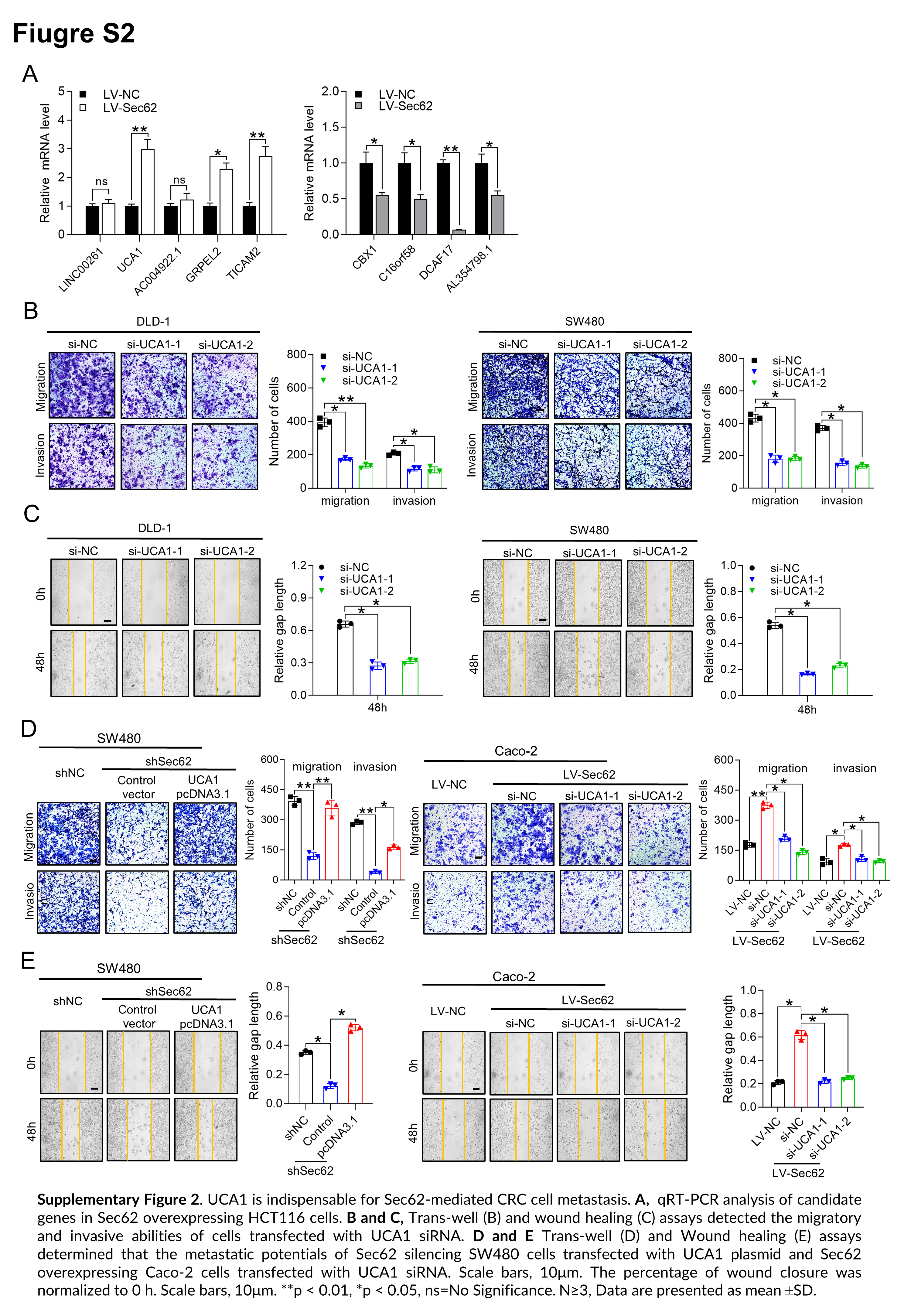

Supplement: Supplementary file 4 — FIGURE S2 UCA1 is indispensable for Sec62‐mediated CRC cell metastasis. (A) qRT‐PCR analysis of candidate genes in Sec62 overexpressing HCT116 cells. (B and C) Trans‐well (B) and wound healing (C) assays detected the migratory and invasive abilities of cells transfected with UCA1 siRNA. (D and E) Trans‐well (D) and Wound healing (E) assays determined that the metastatic potentials of Sec62 silencing SW480 cells transfected with UCA1 plasmid and Sec62 overexpressing Caco‐2 cells transfected with UCA1 siRNA. Scale bars, 10 μm. The percentage of wound closure was normalized to 0 h. Scale bars, 10 μm. **p < 0.01, *p < 0.05, ns = No Significance. N ≥ 3, Data are presented as mean ± SD [file CPR-55-e13253-s002.tif]
